# The Political, Economic and Socio‐Cultural Discourse Surrounding the Backyard Chicken‐Rearing Farming Systems in the Western and North‐Western Provinces of Sri Lanka

**DOI:** 10.1002/vms3.70174

**Published:** 2025-04-15

**Authors:** Mahadura Indrajee Lilantha De Zoysa, Ayona Silva‐Fletcher, Herath Mudiyanselage Amani Sewwandi Herath, Manamperi Muyanselage Shanilki Lochana Yalegama, Ruwani Sagarika Kalupahana, Hatharasinghe Arachchige Sriyani Satharasinghe, Anil Wasantha Kalupahana, Eriyagolla Mudiyanselage Dularika Dananjani Kumari Karunarathna, Kohilawatte Gamage Dona Tharini Layanvi De Alwis

**Affiliations:** ^1^ Department of English Language Teaching Faculty of Arts University of Peradeniya Peradeniya Sri Lanka; ^2^ The Royal Veterinary College University of London London UK; ^3^ Department of Veterinary Public Health and Pharmacology Faculty of Veterinary Medicine and Animal Science University of Peradeniya Peradeniya Sri Lanka; ^4^ Poultry Development Unit Udahamulla Nugegoda Sri Lanka; ^5^ Department of Veterinary Pathobiology Faculty of Veterinary Medicine and Animal Science University of Peradeniya Peradeniya Sri Lanka

**Keywords:** backyard chicken, discourse, political cultural practices, religion, sustainability

## Abstract

**Background:**

The poultry sector is the largest contributor, in terms of the livestock sector to the Gross Domestic Product in Sri Lanka, providing economic security to the country and food security to people. There are three farming systems: broiler, layer and backyard chicken. The backyard chicken farming system is widespread across Sri Lanka. The population in Sri Lanka comprises five ethnic groups, four religious practices and is from a wide spectrum of economic classes.

**Objectives:**

The study was conducted to explore the political, economic and cultural discourse surrounding backyard farming in the Western and North‐Western Provinces of Sri Lanka. The overall purpose is to identify challenges and enablers to make backyard chicken rearing a sustainable economic activity.

**Methods:**

This study used qualitative research methodology. Thirty‐eight semi‐structured interviews were conducted among backyard poultry farmers in two provinces and the data were transcribed, tabulated, coded and themes were generated. Then, data under the ensuing themes were analysed using critical discourse analysis.

**Results:**

The data revealed how backyard chicken rearing and its associated practices, such as feed formulation, issues associated with biosecurity, marketability and sustenance of the sector, are influenced by political, economic and cultural factors, turning it into a complex discursive space.

**Conclusions:**

There are political, economic and cultural factors that may act in juxtaposition in backyard chicken farming in Sri Lanka. The results of this study can be used to underpin policy formulation, taking into account the prevailing political, economic and cultural practices and beliefs of backyard poultry farmers.

## Graphical abstract

Backyard chicken farming is practised widely across diverse communities in Sri Lanka. This study exploring the political, economic and cultural factors affecting backyard chicken farming in the Western and North‐Western Provinces in Sri Lanka aims to identify the challenges and enablers to enhance the sustainability of the sector.

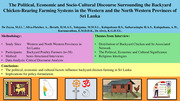

## 1 Introduction

The livestock sector plays a major role in providing economic and nutritional security for people, contributing around 1% to the total Gross Domestic Product (GDP) in Sri Lanka (Department of Animal Production and Health (DAPH), Sri Lanka 2021). Among the key livestock industries operating in Sri Lanka, the poultry sector holds a prominent position, primarily attributed to its substantial contribution to the national GDP and its capacity to generate greater tax revenue when compared to other livestock and fisheries industries (Manjula et al. 2018).

The poultry sector in Sri Lanka ranges from intensive commercial systems to backyard chicken production. In intensive production systems, birds of exotic and mixed breeds are reared under high‐input management with intensive use of capital and labour (Manjula et al. 2018; Samanta, Joardar, and Das 2018; Silva et al. 2014). The intensive system functions under large‐scale operators, and they are highly commercialized market‐oriented systems. In contrast to this, backyard chicken rearing systems consist of indigenous poultry stocks that are ‘allowed to roam freely and scavenge for their feed. It's a low‐risk, low‐investment, low‐production, low‐return enterprise system with an extensive form of management’ (Korale‐Gedara et al. 2018; Silva et al. 2014). Backyard chicken rearing mostly consists of small flocks kept under low or minimal biosecurity measures (Conan et al. 2012; Kumar, Dahiya, and Ratwan 2021) and mostly consists ‘of free indigenous unselected breeds of various ages, with various species mixed in the same flock’ (Conan et al. 2012). Backyard chicken‐rearing is also used as an effective means to improve the ‘socioeconomic and nutritional status among rural poor people of the society due to availability of a cheap source of protein (egg and meat), for eradication of malnutrition, generation of self‐employment and supplementary income’ (Kumar, Dahiya, and Ratwan 2021, 1477). Backyard chickens play a vital role in providing livelihoods to many rural households in the developing world (Idamakoro and Hosuq 2022; Alders et al. 2018). The output of backyard chickens is lower than that of intensively reared birds, ‘but it is obtained with a minimum input in terms of housing, disease control, management and supplementary feeding’ (Alders and Pym 2009, as quoted in Alders, Bagnol, and Young 2010, 434).

Although commercial layer and broiler production systems are the predominant contributors to the Sri Lankan market in terms of supplying eggs and meat through organized industries, the backyard system operates through its own unique methods. Abeykoon et al. (2014) emphasize that the potential of backyard chicken rearing to provide nutrition and enhance income for rural farmers has not been fully realized. It is important to investigate the reasons behind the backyard chicken‐rearing sector's failure to emerge as a profitable industry. In the backyard sector in Sri Lanka, ‘limitations in inputs such as land, feed and breeding stocks were the main drawbacks’ (Abeykoon et al. 2014, 163).

The local backyard chicken‐rearing sector transcends mere economic activity, intertwining with capitalism, markets, income, livelihoods, tradition, culture, religion and beliefs. Rearing backyard chickens becomes a complex discursive activity, representing a space constructed through diverse and politically, socially and culturally charged discourses.

In Sri Lanka, the cultural fabric is mainly made up of Sinhalese, Tamils, Burghers and Muslims and a smaller proportion of indigenous communities. In terms of religion, the majority of Sinhalese follow Buddhism and others follow Christianity, Hinduism and Islam (as shown in Tables 1 and 2).

**TABLE 1:** Composition of population by ethnicity (Central Bank of Sri Lanka 2022).

Sinhalese	74.9%
Sri Lankan Tamil	11.2%
Indian Tamil	4.1%
Sri Lankan Moor	9.3%
Other	0.5%

**TABLE 2:** Composition of population by religion (Central Bank of Sri Lanka 2022).

Buddhist	70.1%
Hindu	12.6%
Islam	9.7%
Christian and Roman Catholic	7.6%

These culturally diverse groups follow different practices and subscribe to different norms and values. These norms, values and beliefs affect the existence of people belonging to different groups as a whole, defining how they engage in a particular economic activity. Rearing backyard poultry is an activity conducted across the spectrum of these cultures and is carried out using different processes and practices. This may lead to various tensions and dissonance.

In order to unearth the discourse and explore the ontological and epistemological perspectives, the research paradigm used was social constructivism. Within social constructivism, the qualitative approach was followed as it would yield non‐statistical data which can be analysed through non‐statistical means because this method will allow multiple stories to emerge without reducing the findings to numerical data (Dornyei and Ushioda 2011). Qualitative research attempts to study ‘human behaviour within the context in which that behaviour would occur naturally and in which the role of the researcher would not affect the normal behaviour of subjects’ (Seligner and Shohamy 1989, 118).

Sri Lanka is slowly recovering from the economic crisis of 2023 (https://www.worldbank.org/en/country/srilanka/overview), and backyard poultry can be promoted as a pragmatic means to reduce unemployment and ensure nutritional security across Sri Lanka. Ongoing work and experience with the backyard poultry sector demonstrate that this is not a business‐oriented venture but has the potential to be so.

The study aimed to explore the political, economic and socio‐cultural discourse surrounding backyard chicken‐rearing farming systems in the Western and North‐Western Provinces of Sri Lanka. The overall goal was to identify the enablers and barriers to making backyard chicken rearing a successful economic activity in the country.

In this manuscript, the term ‘political’ means the elements of power, how power operates and the power relations that exist between the different entities discussed in this manuscript. For example, the different power dynamics between the veterinarians and the farmers in trying to get the farmers to adhere to vaccination schedules, biosecurity practices and regulations are discussed in the manuscript. By ‘economic practices’, we refer to the variables dealing with the income or expenditure. For example, in terms of income, the income generated through selling eggs, meat and birds for other purposes is discussed in the article. In terms of expenditure, the costs incurred in the physical construction of chicken coops, sourcing feed and sourcing veterinary medicines are explored in the manuscript. By ‘socio‐cultural aspects’, we directly study various cultural elements in terms of religious beliefs, practices related to black magic and other various local practices associated with backyard chicken rearing in Sri Lanka.

## 2 Research Methodology

### 2.1 Study Sites

**MAP 1:**
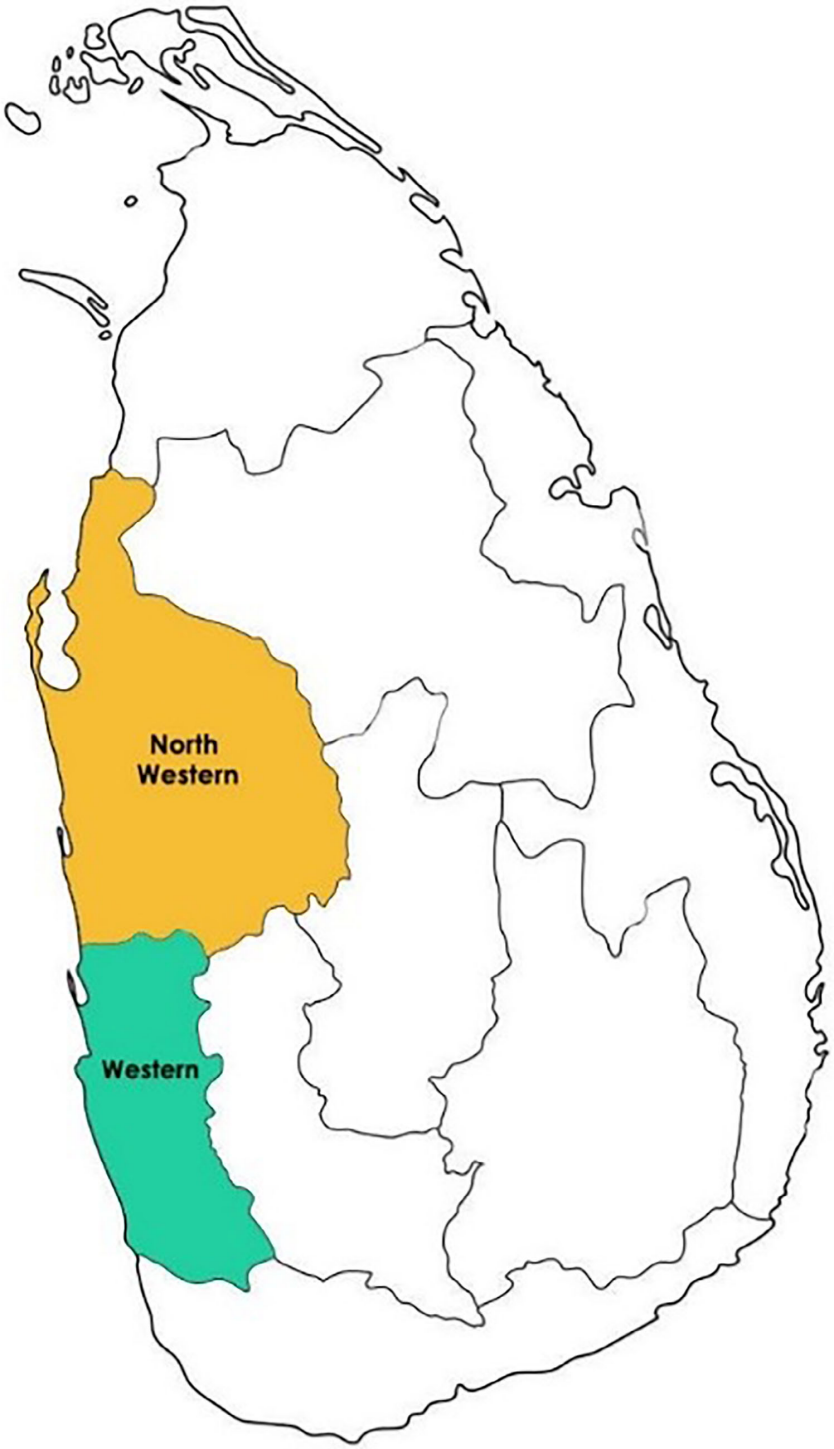
A map of Sri Lanka denoting the two provinces (Western and North‐Western) selected for the study.

Sri Lanka consists of nine provinces. Two provinces, namely, the Western Province and North‐Western Province, were selected on the basis that they had the highest poultry density across Sri Lanka (Central Bank Report, 2022), and data were collected to cover the respective districts of these two provinces (as shown in Map 1). The Western Province, also known as the commercial hub of Sri Lanka, consists of three districts, namely, Colombo, Gampaha and Kaluthara. The poultry densities of these three districts are 4826.63, 1597.84 and 780.60/km^2^, respectively. The North‐Western Province, which consists of two districts, Kurunegala and Puttlam, is the other poultry‐dense geographical space in Sri Lanka. The poultry densities of Kurunegala and Puttlam are 1522.87 and 1291.09/km^2^, respectively (Table 3).

**TABLE 3:** Poultry density in Western and North‐Western Provinces in Sri Lanka (Central Bank of Sri Lanka 2022).

District	Area (km^2^)	Number of chickens	Poultry density (number of chickens/km^2^)
Colombo	699	3373,820	4826.63
Gampaha	1387	2216,210	1597.84
Kalutara	1598	1247,400	780.60
Kurunegala	4816	7334,170	1522.87
Puttlam	3072	3966,230	1291.09

Though data about the number of registered backyard farms are available in reports produced by the Department of Census and Statistics and the Department of Animal Production and Health, they do not contain information about the number of birds in a farm or the poultry density across Sri Lanka. It must be stated that the number of backyard chicken‐rearing farms is not an indication of the number of birds or their density. Likewise, the statistics present on backyard chicken‐rearing farms do not represent whether these farms are operative or not because backyard chicken‐rearing is not a well‐organized economic activity in Sri Lanka. Therefore, the most poultry‐dense districts were selected based on the statistics presented by the Central Bank of Sri Lanka due to their reliability and validity. In addition to this, chicken density was used as a criterion, as marketing and food channels are readily available and are in operation in these provinces. This is essential for backyard farmers to obtain food and sell the products. Moreover, farmer associations, farmers, information about farming and data about farming activities are readily available as opposed to other provinces where chicken density is low. The ethnic and religious spread of people is another reason for selecting these provinces, which is relevant to the study.

### 2.2 Participants

The interviews were conducted with 38 backyard chicken farmers (as shown in Table 4) who were selected on a convenience sampling basis. These participants were selected with the assistance of the divisional veterinary department through the registry they maintained with the support of the veterinarians employed in the particular province. Table 5 presents the inclusion and exclusion criteria used in selecting the farmers for the study. The same inclusion and exclusion criteria were used in both provinces.

**TABLE 4:** Participants of the study.

Province	District	Respondent code	Number of chickens	Gender	
				Female	Male
Western	Colombo	W1	500		
		W2	180		
		W3	30		
		W4	50		
		W5	15		
		W6	10		
		W7	100		
		W8	20		
	Gampaha	W9	50		
		W10	12		
		W11	12		
		W12	100		
		W13	42		
		W14	15		
		W15	40		
		W16	20		
		W17	50		
	Kalutara	W18	10		
		W19	15		
		W20	15		
		W21	6		
		W22	30		
		W23	30		
North‐Western	Kurunegala	NW1	25		
		NW2	150		
		NW3	150		
		NW4	130		
		NW5	39		
		NW6	35		
		NW7	80		
		NW8	5		
		NW9	240		
		NW10	50		
		NW11	10		
		NW12	100		
	Puttalam	NW13	30		
		NW14	10		
		NW15	05		

**TABLE 5:** Inclusion and exclusion criteria of the study.

Inclusion criteria	Exclusion criteria
The participants should be strictly backyard poultry farmers	Participants who reared commercial poultry were excluded
They should rear more than 5 birds	The farmers who had more than 500 birds were not included in the study
They should be residents of the two provinces taken for the study	Non‐residents of the provinces were excluded
They should be engaged in active farming	Those whose farms had closed down or those who were not engaged in active farming were excluded

The interviews were conducted in the province they were domiciled in, within their own farms. The participants were not requested to come to any other location or a different location. The participants were first given an introduction to the research and its objectives. Their consent was then gained, and permission was also taken to audio‐record the interviews. They were also informed that they could opt out of the interview at any given point in time, but none of the participants did so. Interviews were conducted by two of the principal investigators while the other researchers took down notes and observed the scenario. The interviews were carried out in the participants’ preferred language. A single interview lasted from 30 to 35 min. The participants were also told that they could view their interview transcripts if they wished, but again no such requests were made. To avoid interviewer bias, we asked the same questions of those who were interviewed. This process was facilitated further as the researchers used a semi‐structured template containing the questionnaires. After a considerable number of interviews, similar rhetoric began to emerge from participants, and then it was understood that data saturation had been reached. Therefore, the interviews after that were stopped.

### 2.3 Data Collection Techniques

The research was conducted through a qualitative approach. When carrying out research based on discourse studies, the qualitative approach will allow the researchers to explore the phenomena studied in an in‐depth manner. Semi‐structured interviews were used as the research/data collection tool because this will allow the participants to state their perceptions without hindrance. Thus, the interview guide consisted of a number of main questions and several sub‐prompts which were developed by the research team covering the dimensions of backyard poultry farming in relation to Sri Lanka. The interview guide consisted of registration information, vaccination techniques and medication, economic assistance, awareness of farmers related to poultry farming, challenges faced by farmers, disease conditions, bio‐security‐related issues, female empowerment and the religious and socio‐cultural practices associated with chickens. The interview guide was then piloted in the two chosen districts, and necessary amendments were made. New prompts were added according to the feedback received, and some other minor adjustments were also made. Then the actual interviews were carried out on a face‐to‐face basis, and they were carried out in Sinhala. The interviews were carried out within a period of 6 weeks.

### 2.4 Data Analysis

The audio recordings were initially transcribed in Sinhala and then translated into English by the main researcher. The second researcher confirmed the contents of the translation. The translated transcripts were then re‐read several times, and the emergent codes were tabulated (Saldana 2009). The recurrent codes were used to generate themes. The emergent overarching themes were the distribution of backyard chicken and its associated network, feed practices, biosecurity, the political, economic and cultural significance of backyard chicken and lastly, the religious ideologies associated with backyard chicken. These themes were validated and verified. This process of validity was ensured by following an intercoding reliability process where all the researchers independently coded a sample of transcriptions. These codes were compared, and a level of agreement was reached. The researchers then agreed on the emerging themes. This ensured that the coding process was consistent and could be applied reliably by different individuals. The headings of the results section are organized under the themes that emerged from the data analysis. The data coming under these themes were then analysed by the researchers using critical discourse analysis (Fairclough 2013; Wodak 2014), and deconstruction was used as the major theoretical formulation when conducting the analysis.

## 3 Results and Discussion

### 3.1 Distribution of Backyard Chicken and Its Associated Network

The rearing of backyard chickens is neither a structurally organized system based on public/private administrative principles nor a structurally state‐governed system. But this is not to state that the system is structureless, as it has its own modus operandi developed through years of locally developed practices. But without serious techno‐scientific interventions or administrative structuralizations, rearing backyard chickens has survived for ages.

One‐month‐old chicks are generally distributed freely among interested parties through the divisional veterinary offices under various government‐aided projects for backyard chicken farmers. The month‐old chicks are purchased by the veterinary offices from the Karandagolla (Karandagolla farm, also known as the Central Poultry Research Station, is located in Kurunegala District, North Western Province, Sri Lanka) or Kotadeniyawa (Kotadeniyawa farm is in Gampaha District, Western Province, Sri Lanka) breeding farms. If not, some private individuals who own hatcheries sell day‐old chicks to interested parties. These hatcheries operate at various levels, where some use imported egg hatching machines while others use locally sourced and locally produced hatching machines made from disused refrigerators or Rigifoam (rigid foam sheets made from polystyrene) boxes. Hatchery owners at times source eggs from selected backyard farms or their own farms for hatching purposes.

I get the eggs and sell them to a hatchery owner who comes from Marawila. The rest I sell to the shops. (Respondent NW2, Field Interviews, 2022)

Likewise, there are a small number of individuals who hatch eggs using natural methods by allowing hens to incubate naturally. Primarily, these eggs find their market among neighbours and interested individuals who either directly purchase from the farm or through sales at nearby shops; prices range from LKR. 30–35 (0.08–0.09 USD)/chick as of 2022 (Supporting Information).

The farmers also sell the backyard chicken for meat, mostly to intermediaries who then take them, slaughter them and sell them in the open market. Prices of chicken vary depending on their size and gender. In 2022, a rooster was sold for approximately Rs. 1500 (4.08 USD), and a spent hen was sold for around LKR. 600–800 (1.6–2.17 USD). In layman's terms, as respondent W1 pointed out,

Chickens are like ATMs (automated teller machines or cash machines). Whenever you want cash, you can almost surely sell them without much of a problem. (Field Interviews, 2022)

Samanta, Joardar, and Das (2018) also express a similar sentiment by stating that backyard chicken rearing will provide ‘ready cash in times of hardship or emergency, which may make the difference between life and death’ (p. 481). In addition, some farmers also rear game fowls and pointed out that:

rearing them is not illegal but using them for fights is. If these birds win fights, their prices go up to around Rs. 300000‐Rs. 400000 (817.43–1089.91 USD) which is quite something. If they don't win games, we can anyway sell them for a good price. (Respondent W7, Field Interviews, 2022)

#### 3.1.1 Feed Practices

When discussing the feed practices followed in backyard chicken‐rearing systems, backyard chickens will survive on household food scraps, kitchen vegetable waste and green grass and will source their own feed with little supervision (Abeykoon et al. 2014; Alders, Bagnol, and Young 2010; Kumar, Dahiya, and Ratwan 2021). The validity of these observations and statements depends on several decisive factors such as the size of the flock and the space/location the farm is set in. Furthermore, backyard chickens’ genetic makeup also plays a role in determining the feeding practices.

If the flock of birds ranges from 5 to 10 birds and the backyard farm is set in a spacious location with a substantial availability of forage, the chickens can be left alone to scavenge for food on their own:

I let them out in the morning and they find their feed. We do not need to feed them anything much. We have a fairly large land, so they roam around and eat and come back home around 5.00 p.m.–6.00 p.m. every day. (Respondent NW9, Field Interviews, 2022)

The same idea was iterated by respondents whose flock did not exceed 5–15 birds, depending on the location (i.e., the availability of land) in which their farms are situated. These farmers were not worried about the number of eggs the chickens produce, as most of the eggs are used for domestic consumption, and whatever is left would be either given to neighbours or sold off to a shop.

But problems arise when commercial interests are put forth, especially in terms of increasing the number of birds in order to receive a higher yield of eggs and meat. Then, if the land area in which the farm is situated is not large or spacious enough, which is now the case across Sri Lanka due to rapid urbanization, the chickens do not have enough grounds, or anything left in the grounds to scavenge from.

[d]uring those days, when the number of chickens was limited, they could find their own feed. But when the flock size increased, there was nothing for them to feed on in the land and look, the area of the backyard farm and my land is also small. (Respondent W3, Field Interviews, 2022)

Therefore, it becomes evident that the only solution to providing the chickens feed would be to give them commercial feed that is available in the market. Many farmers who had a considerable number of birds beyond 20 stated that they give the birds commercial feed in the form of both grower and layer feed. Besides the regular feed, an array of supplementary vitamins is administered to the chickens. This practice aims to enhance egg production and promote optimal physical development in the chickens, ensuring they can be sold at a favourable price. Likewise, it was also pointed out that the

new breed of backyard chickens, now genetically intermixed, require commercial feed in order for them to lay eggs. (Respondent W16, Field Interviews, 2022)

The problem with this is about buying commercial feed directly from the market. Due to the COVID‐19 pandemic and the economic crisis in Sri Lanka, the price of commercial feed has increased by excessive amounts and/or is not available due to import restrictions. As commercial feed is not provided on a subsidy scheme to the farmers by the government or any other government agency, backyard chicken farmers who have started to rear a large number of birds have started to face severe difficulties in sourcing the required feed to keep the flock alive and productive:

[w]ith the rise in poultry feed prices, it is hard to find money to buy feed. And it is reducing our profit margins too. We have to increase the price of eggs and meat both if so and also make sure egg production is more (Respondent NW6, Field Interviews, 2022).

To overcome this issue, government veterinary surgeons have been promoting a scheme of making your own feed at home, which can then be given out to the chickens:

We were instructed by the doctor to take two empty paint buckets, cut the bottom of one and keep that one on the ground. Then we were told to put the other bucket on top of it and to put kitchen waste into the bucket and keep it for two to three days. The doctor said that within two to three days, worms would emerge and that we could feed them to the chickens and we were also told not to feed the chickens with kitchen waste just like that. But will that be enough and will they lay eggs? We are not sure. (Respondent W1, Field Interviews, 2022)

Furthermore, despite such advice, many of the farmers either had not started to follow the practice or were even reluctant to start it. When questioned why, they merely pointed out that they should start it in the time to come. Their reluctance itself alludes to the fact that they trust commercial feed over homemade feed and that they are unsure whether it would increase their chances of gaining a profit.

#### 3.1.2 Biosecurity Practices

Another interesting aspect of backyard chicken rearing is the adherence, or lack thereof, to biosecurity practices. Backyard chicken production is characterized by low biosecurity measures or the lack of adherence to biosecurity measures as propagated through Western scientific medicinal practices (Conan et al. 2012; Correia‐Gomes and Sparks 2020; Samanta, Joardar, and Das 2018). Biosecurity is defined as measures or safety practices which should be followed or adhered to by the farmers to prevent the entry of diseases into and out of the chickens to other animals and humans, which will improve both human and animal health (Samanta, Joardar, and Das 2018). Therefore, biosecurity is ‘an established international policy concern, with its own terminologies, governance structure, and expert science base’ (Maye et al. 2012, 150–151).

Nevertheless, these measures are not followed due to a lack of knowledge (Correia‐Gomes and Sparks 2020) or lack of capital (Samanta, Joardar, and Das 2018), which makes the uptake of the ideals presented by biosecurity very low (Bleich, Pagani, and Honhold 2009). It is interesting to note that many backyard chicken‐rearing farmers, despite attending training programs, do not know what the term biosecurity means at a conceptual level.

All the respondents pointed out that they had not heard of such a concept. Most of them wore no special clothing when entering the chicken pen and most of the farmers wore mere sandals (rubber slippers/flip flops) or did not wear any form of footwear. There were no mechanisms in place to wash the hands after exiting the pen, and such behaviour was not observed:

I know we have to wear boots, use chemical baths and wash our hands, but if we are to do all that, the cost will go up exponentially. We cannot raise the price of eggs or meat to match the cost. (Respondent W13, Field Interviews, 2022)

Therefore, farmers, despite not knowing the term biosecurity or its conceptual background, know that practices to safeguard themselves and the flock should be followed. The only reason why most of them cannot implement such strict practices is due to the lack of capital. If they do spend, as shown by respondent W13, they would have to increase the price of eggs and meat, which would lead them to lose their market share or the customers.

In terms of structural biosecurity, all the backyard farmers either had chicken coops built up of bricks or wood but had no proper biosecurity fencing in place. The non‐adherence to structural biosecurity practices has led to an increase in baseline mortality due to predators such as stray dogs, stray cats, a bird called *kurulu goya*, mongoose, pole cats, snakes (mostly rat snakes and cobras) and people who steal the chicken.

Thus, many backyard chicken‐rearing farmers have initiated a simple biosecurity practice in the form of rearing dogs in the house to protect the flock:

We just keep two dogs at home on either side of the pen. They chase away most of the predators or at least bark. They don't harm the chickens we keep at home. (Respondent NW2, Field Interviews, 2022)

Some farmers have adopted specific behaviours to protect their chickens.

I mostly wait outside when I let these chickens out and spend that time watering the plants or doing some work in the garden so that I can keep an eye on them. (Respondent NW5, Field Interviews, 2022)

Other than structural biosecurity measures, another biosecurity measure is adherence to vaccination protocols. It is stated that improper adherence to vaccination schedules can lead to ‘infectious diseases (e.g., Newcastle disease (ND), salmonellosis, Gumboro disease or fowl typhoid)’ (Conan et al. 2012, 2). In Sri Lanka, there is no set vaccination protocol followed by the regional veterinary offices. Thus, different areas have different protocols for vaccinating day‐one‐old chicks. According to respondent W12, generally, Marek's, Ranikhet (Newcastle disease), Gumboro and Fowl Pox injections are administered together with deworming. In addition to the above, backyard chicken‐rearing farmers are advised to administer Ranikhet (Newcastle disease) and Fowl Pox injections at least once a year and administer deworming medicine every 2 months (Field Interviews, 2022).

But, due to several reasons, this system is not followed properly or systematically. Injections like Ranikhet (Newcastle disease) are given out to farmers who are registered at the regional veterinary office free of charge. They are given a date and a time when they have to come with a container and ice to take the injections back home and they are given instructions on how to administer the vaccines to the chickens. However, challenges exist:

It is hard to find time and the money to go at times. In addition, sometimes we have to travel 20 odd km to get to the office. That is difficult. (Respondent NW1, Field Interviews, 2022).

Likewise, there are backyard chicken‐rearing farmers who are not registered at the government veterinary office, and therefore, they do not get the vaccines for free. Due to the price of vaccines, these farmers tend to overlook the vaccination schedules.

Other than this, ideologically, backyard chicken‐rearing farmers in Sri Lanka are of the strong belief that backyard chickens, whom the Sinhalese farmers call *Sinhala Kukullu*, that is, ‘Sinhalese Chicken’, are less prone to diseases and that they rarely fall sick, unlike broilers or layers:

[t]hough they say give this vaccine and that vaccine, there is no necessity to do so. Backyard chickens are not like broilers, they are strong and won't get sick. (Respondent W21, Field Interviews, 2022)

Samanta, Joardar, and Das (2018) point out that ‘indigenous or native or nondescript breeds are preferred for backyard farming due to easy availability, higher adaptability to the local environment [and] resistance to some diseases’ (p. 484). Though this stands true, there were instances where respondents pointed out that their chicken died, especially during the COVID‐19, pandemic without access to proper medication. But when the birds fall sick, the farmers have their own means of dealing with it.

These chickens mostly get phlegm or an eye disease. We know they are sick because they stay in one place (*hobbagena/mukagena innawa*) and if it is phlegm, we can hear a wheeze coming in from their throats. So if that is the case, we isolate the chicken and give it two panadol, one piriton and an amoxicillin and they get better quickly. (Respondent NW11, Field Interviews, 2022)

When inquired about consulting a veterinary surgeon, it was noted that they occasionally seek veterinary advice if available. In cases where accessibility is limited, they resort to personal experience, consult pharmacists or, on occasion, approach hatchery owners for guidance. This clearly posits a problem because this alludes to the wide use of antibiotics without a prescription or even a proper clinical diagnosis, which can then lead to the development of antibiotic and antimicrobial resistance. Some farmers do not subscribe to Western medication purely on the grounds that they are too expensive or that they do more harm than good. Some farmers are confident that this is not necessary:

We need additional money to do all that. What we have been doing has not gone wrong yet. (Respondent NW4, Field Interviews, 2022)

The same respondent pointed out that,

we use a lot of indigenous medicine. For example, we grind up ginger, garlic, coriander and pepper along with bird's eye chillies into small balls and feed the chickens. It works like a charm for both phlegm and stomach upsets. (Field Interviews, 2022)

This was a widespread practice, in the areas, away from the urban centres, especially in the North‐Western Province:

we as a practice add one tablespoon of arrack (distilled spirit made from coconut ‐*gal arakku*) and mix it with bird's eye chillies (*kochchi miris*). (Respondent NW14, Field Interviews, 2022)

Again, the validity or the effectiveness of such practices remains to be open and requires more in‐depth studies. The primary factor compelling them in this direction is the deficiency in capital and the absence of a well‐structured system linking farmers with veterinary surgeons, livestock development officers and other regulatory bodies. Such a system is crucial in providing practical assistance concerning disease prevention and control. Many of the farmers were unaware of zoonoses. In Sri Lanka, backyard chicken, turkey, quails (*Watu Kurullo*), cattle, pigs and goats are reared together. Most farms had either chicken and cattle or chicken with goats. Therefore, in the event of a disease outbreak, the ability of it to spread to a large number of different species is very limited.

But, despite these issues, which mainly arise from limited financial capacity, respondents stated that they would like to expand their business if help is granted from the government authorities.

### 3.2 The Political, Economic and Cultural Significance of Backyard Chicken

Alders, Bagnol, and Young (2010) point out that ‘[b]ackyard chickens are active in pest control, provide manure which can be used as fertilizer, *are required for special festivals, and are essential for many traditional ceremonies* [emphasis added]’ (p. 443). This statement stands true in relation to Sri Lanka as well. Native chickens possess a huge symbolic value within Sri Lanka. The chicken is used as an auspicious symbol on top of the oil lamp, which is traditionally lit at the start of ceremonies and functions as a mark of good luck.

The backyard chickens are also used for various black magic, voodoo rituals and cultural activities across Sri Lanka called *Bali Thovil* or *Shanthi Karma*. Foster (1998), quoting Glick (1967), points out that in many cultures, various practices and ideas in relation to illnesses are inseparable from religious beliefs, and thus, the *kukul billa* or the chicken as an offering to a particular god is given under two rituals as to redeem a *bára* and to expel/eliminate illness or evil forces from the human body. ‘The *bára* is a vow made by any person to a deity promising him a certain ritual, feast or another recompense in his honour, if the wishes of the person are granted […] Redeeming the bára, is known as *bára oppu kirima*. Until the redemption of the vow, one is in charge of the deity concerned; if the vow is not discharged, serious misfortunes (*dosa*) may result’ (Obeysekere 1984). Country chickens are sacrificed to deities, such as Goddess *Kali*, *Kalu Kambili*, *Kadawara* and *Devol*, to redeem báras.

The chicken is offered to *kali maani* as an offering. They belong to the Yaksha (devils) clan. They always want such offerings. For example, if somebody steals something that belongs to you, they come to the god and ask for help. If they find what was stolen, they offer a chicken as gratitude. (Respondent NW4 Field Interviews, 2022)

In relation to expelling or eliminating illnesses or evil forces, in rural villages in Sri Lanka, *bali thovil* are conducted for either curing diseases or eradicating evil influences caused due to people's planetary positions. A chicken is used in those kinds of exorcistic ceremonies as a sacrificial animal (*kukulu billa*) to take over the evil (*vas*) of the affected person (Obeyesekere 2022; Kariyawasam 1998).

When you conduct a *thovil*, you need to offer *suniyam* and that requires a backyard chicken. When we offer a chicken, the problems/illnesses associated with the human being go to the chicken. The issues associated with our planetary positions are put on the chicken, we offer a life for a life and that is where the chicken comes in. Therefore, the evil forces fall on the chicken and it dies most of the time. (Respondent W15, Field Interviews, 2022)

But with the influence of Buddhism, ‘with its doctrine of ahimsa, applicable to animal sacrifice, was responsible for the development of the ritual from an actual sacrifice to a symbolic one. If this is so, we see in today's ritual a symbolic displacement’ (Obeysekere 1984). Chickens, now, are rarely killed in these ritualistic ceremonies, but a few drops of blood (*le gotu*) are given to the demon as a substitute for the whole bird. Respondent NW4 stated that ‘he doesn't either kill the animals or take blood from them, but nominally sacrifice them to the God and allow them to stay in the *Devala* (temple) premises’ (Field Interviews, 2022).

Other than these *Bali/Thovil/Shanthikarma* practices, Sinhalese‐Buddhists also use backyard chickens for a ritual known as *ulu ahu pannima* (cross the threshold of a house):

When a person constructs a new house, before entering the house first, they send a chicken across the house from the main entrance to the back entrance. This is done with the hope of removing the evil eye and it is said that if there are evil forces around the house, it will fall upon the chicken and that the chicken dies within seven days if that is the case. Usually, after the chicken walks, the mason who built the house will take the chicken, cull it and eat it. Some do not do so and return the chicken. But mostly, they are killed and eaten. (Respondent W21, Field Interviews, 2022)

Backyard chicken‐rearing farmers, especially Sinhalese‐Buddhists, are reluctant to sell or give the birds for the practices outlined above:

I never sell chicken for such practices. But at times, people will come and buy on the sly without stating the purpose. If that is the case, there is no way we will know. (Respondent W10, Field Interviews, 2022)

Those who do sell chicken sell it for around LKR. 5000 (13.62 USD) for both *bali/thovil* and *ulu ahu pannima*. Hence, the market in all reality is ripe for such activities, but there is a deep‐rooted reluctance to supply birds for such purposes. After the ceremony is over, the farmers never accept the birds and put them back with the flock, stating that

chicken will have the evil forces bonded to him. So, that will affect the other chickens in the flock too. (Respondent NW4, Field Interviews, 2022)

Respondent NW3 narrated these incidents during the course of the interviews:

Once I gave a chicken for a *thovil* and once the *thovil* was over, they brought the bird back to me. They had just pricked the chicken wattles and just taken a few drops of blood. The poor fellow was okay. But after it was brought in, I fell sick within a couple of days. For weeks I was sick and medicine did not work. I thought there must be something wrong and remembered the incident about the chicken. I told my son to take it away from the house and he took it off. Within a day, I got better. (Field Interviews, 2022)

Though there is no rational explanation for the above sequence of events, it just shows the mindset of the Sri Lankan villagers who subscribe to such culturally and scientifically contested ideologies and ideals.

Sin or not I do not know, but these chickens end up in the Athuraya's (the shaman carrying out the Thovil Ritual) and his assistant's stomach. (Respondent W17, Field Interviews, 2022)

If the chicken is not culled or the previous owners reject taking it back, they are mostly donated to the temple or a *devalaya*. Before the bird is taken in by the temple or the *devalaya*, the *bandanaya*, that is, the evil bind, is ‘cut’ by the *Kapurala* (priest) after conducting a small religious ceremony (pooja), and then the chicken is allowed to roam freely in the temple (*devala*) premises. Despite residing in what is considered an advanced post‐modernist era, such practices continue to permeate the cultural landscape in Sri Lanka. The animals, who, at the end of the day, have no right to their lives and are used to fulfil the desires of human beings. Especially in moments when language goes on holiday, that is, when language is unable to provide a rational explanation as to why an incident occurred at a particular point in time and place (Wittgenstein 1933), voodoo and black magic practices become prominent.

### 3.3 Religious Ideologies Associated With Backyard Chicken

Religious doctrines, ideas, beliefs and value systems in human societies guide and influence the way their members act, even in the economic sphere. Religions dictate certain preferred guidelines of behaviour according to which their followers orient their activities.

In the ideologically laden context of Sri Lanka, Buddhism as a religious doctrine plays a key role in shaping the socio‐economic behaviour of Sri Lankans. This notable presence of Buddhism in the social fabric of Sri Lankan society is one major factor which influences the economic decision‐making of farmers engaged in backyard chicken rearing.

In terms of religious ideology, Sinhalese Buddhists resist culling chicken for the purpose of selling for meat. The average Sri Lankan mindset is still far etched and concretized in the religious cosmos where the notion of Karma and Sin plays a huge role. Malalgoda (2023) states that one of the dilemmas affecting Buddhism is the ‘practical inaccessibility of the path to salvation’ (p. 15) and making it available to human beings in general as this entailed renunciation of all worldly pleasure. Although renunciation was favoured by certain ‘radical’ monks, for the laity, there existed a gap between salvation and the need to achieve it, which could go on for years and years of rebirth. Thus, as Malagoda (2023) shows, the laity was asked to engage in merit‐making (*Punna kamma*) to fill that gap, and as they were not ‘morally strong enough to transcend all desires, were prescribed to cultivate the desire for meritorious deeds, so that they could improve their chances for better rebirths’ (p. 15). Hence, killing a chicken, for many Sri Lankans, comes across as bad karma within this religious cosmos, which then, according to the religious myth, might affect their well‐being during their current life, afterlife and even their next birth:

It is a sin to kill these chickens. It will bring us bad karma. And they are like family members. When my child or I go inside the cage, or we go close to them, the chicken climbs on top of our shoulders even. They are very close to us and are like our family. (Respondent NW5, Field Interviews, 2022)

For some farmers, chickens are part of the family:

I rear these chickens as my children now. My children live away from us now. So, I just do this not to earn money or for the purpose of profit but to take my loneliness and sadness away. So, there is no way I can kill them for the purpose of selling them for meat. (Respondent NW2, Field Interviews, 2022)

For some, chickens must be looked after until they die a natural death:

The doctor always tells us that we should sell the chicken for meat without thinking about karma or sin. But how can we do so? I was told that giving the chicken for meat would bring in good karma because I would help feed another mouth. And I was also told that when the chicken gets old it will suffer because it cannot eat or drink water and that I will have to tend for it daily. Even though they say so, I won't kill the chickens or sell them to be killed. As long as they will live I will feed them and try to look after them. It is a sin to kill. (Respondent W22, Field Interviews, 2022)

Therefore, expert advice on profit‐oriented backyard chicken farming becomes contested on cultural grounds. Even in relation to vaccination and disease prevention, the responses of the farmers are the same, where they do not necessarily subscribe to expert advice purely on the grounds that such practices are not necessary within their contexts. Hence, western scientific knowledge on rearing practices given as knowledge by the veterinary surgeons or the veterinary establishment tends to be contested on the basis of local rearing and local knowledge paradigms influenced especially through religion:

I am going to close down the farm and sell the chickens. The chief prelate of the temple I go to asked me why I am so interested in accumulating bad karma at this old age. I am one of the main patrons and he told me that it is not befitting of me to engage in chicken farming. (Respondent NW10, Field Interviews, 2022)

Therefore, backyard farming becomes a very conflictual, discursive, politically charged space. But as stated before, these local belief systems and rearing methods and the paradigm as a whole are contested by the veterinary establishment by stating that these are pre‐modern age‐old constructions that are detrimental to the survival and performance of a farm.

These pervasive religious ideologies are coupled with the notion of considering chicken as part of the family, which was discussed previously (Desta, 2021; Alders et al. 2018). On one hand, there is a belief that slaughtering chickens may attract negative karma, whereas on the other hand, these birds are regarded as pets, extensions of the family and, in some cases, even as children. These cultural norms and behaviours will affect the productionist paradigm of the poultry sector and will ultimately affect the notion of profit and profitability, which is very important within the neoliberal economic order. Not only does it bring forth paradigms related to production, but it also affects consumption as well.

I have stopped eating both meat and eggs because I see these chickens all the time. (Respondent NW5, Field Interviews, 2022)

Five respondents from both provinces also stated that they do not consume meat or eggs from the chicken they own but they give eggs to their children. But other than the pure vegetarians, most respondents consume meat from other chickens and serve chicken to visitors or other occupants of the household. As Alders, Bagnol, and Young (2010) point out, ‘In many countries, social goodwill is created by offering guests a meal containing meat, more of than not, poultry’ (p. 434).

## 4 Conclusion

Distribution of backyard chickens and their associated network, feed practices, biosecurity practices, political, economic and cultural significance of backyard chickens and religious ideologies associated with backyard chickens discussed above play an important role in backyard chicken‐rearing systems in Sri Lanka. From a positivist perspective, backyard chicken rearing may be perceived simply as an economic activity, offering employment opportunities and a method to enhance nutritional security. As a result, both state and non‐state actors engage in various interventions with the aim of promoting backyard poultry within the community. But this space has become a contested discursive space where complex political, economic and cultural variables interplay. This leads to the creation of an interesting dichotomy and a divided consciousness among the people who are willing to and those engaged in backyard poultry farming. On the one hand, the respondents want to expand their business and expect government help. Yet, there is resistance to producing chicken meat by farmers, especially Sinhala Buddhist farmers (as in the sample taken), on religious, cultural and personal grounds. Through farmer training programmes, it is possible to address the issues associated with feed and biosecurity practices. But it is questionable whether such programmes could in reality bring forth an ideological revolution to change the religious mindset with regard to chicken rearing of the people concerned. As evidence tends to support that chicken rearing can provide the much‐required nutrition for communities, it is imperative to secure the sustainability of this dynamic sector, considering its political disjunctions and discursivities, and to identify sustainable measures ensuring its ongoing viability. The results of this study will be useful in deciding future research avenues and formulating policy. For example, how religion affects the outlook on the poultry sector is a possible future research avenue which will have wider policy implications. Results can also be used to conduct further research on whether livestock should be promoted in certain areas in the country as opposed to the others, which again can have policy implications. Identifying the enablers and barriers to biosecurity practices can also pave the way for further research and have significant policy implications. The overall goal was to understand the factors that enable or hinder the success of backyard chicken rearing as a sustainable economic activity in the country, and some aspects of this have been achieved through this study.

## 5 Limitations of the Study

The study has several limitations. First, the data were not analysed through a gendered lens, which would have broadened the scope of the study. In addition, the study's findings are primarily based on qualitative data, highlighting the need for further quantitative research to provide additional validation and a more comprehensive understanding of the research area.

## 6 Future Research

This research specifically aimed to explore the political, economic and cultural discourse surrounding backyard chicken‐rearing farming systems with the goal to identify the enablers and barriers to making backyard chicken‐rearing a successful economic activity in the country. In order to gain an understanding in depth, it was necessary to take an interview‐based qualitative approach. This is not possible through quantitative methods such as questionnaires. However, to study the prevalence of some practices and behaviours, a questionnaire‐based study is necessary. Moreover, using in‐depth qualitative interviews before conducting questionnaire‐based surveys is more effective in understanding complex issues in a local context. Therefore, a mixed‐methods approach using qualitative and quantitative research tools can be useful. However, mixing methods has several challenges, including the mixing of epistemological and ontological viewpoints and the challenges to researchers in terms of skills and time. It may be the case that a follow‐up study, with a wider aim, could be conducted to identify the prevalence of these views within Sri Lanka and other countries.

## Author Contributions

*Study concept and design*: Ruwani Sagarika Kalupahana, Mahadura Indrajee Lilantha De Zoysa, Herath Mudiyanselage Amani Sewwandi Herath, Ayona Silva‐Fletcher and Hatharasinghe Arachchige Sriyani Satharasinghe. *Data collection and transcribing*: Mahadura Indrajee Lilantha De Zoysa, Herath Mudiyanselage Amani Sewwandi Herath, Manamperi Muyanselage Shanilki Lochana Yalegama, Eriyagolla Mudiyanselage Dularika Dananjani Kumari Karunarathna, Kohilawatte Gamage Dona Tharini Layanvi De Alwis and Hatharasinghe Arachchige Sriyani Satharasinghe. *Analysis and interpretation of data*: Mahadura Indrajee Lilantha De Zoysa, Herath Mudiyanselage Amani Sewwandi Herath, Ruwani Sagarika Kalupahana, Anil Wasantha Kalupahana, Ayona Silva‐Fletcher, Manamperi Muyanselage Shanilki Lochana Yalegama, Eriyagolla Mudiyanselage Dularika Dananjani Kumari Karunarathna and Kohilawatte Gamage Dona Tharini Layanvi De Alwis. *Drafting of the manuscript*: Mahadura Indrajee Lilantha De Zoysa, Herath Mudiyanselage Amani Sewwandi Herath, Ayona Silva‐Fletcher, Ruwani Sagarika Kalupahana and Anil Wasantha Kalupahana. *Critical revision of the manuscript for important intellectual content*: Ayona Silva‐Fletcher, Ruwani Sagarika Kalupahana, Anil Wasantha Kalupahana, Mahadura Indrajee Lilantha De Zoysa and Herath Mudiyanselage Amani Sewwandi Herath. *Obtained funding*: Ayona Silva‐Fletcher and Ruwani Sagarika Kalupahana. *Agreement of the Authors*: All the authors agree to be accountable for the contents of this research article.

## Ethics Statement

This study was approved by the ethical clearance committee, Faculty of Medicine, University of Peradeniya, Sri Lanka under Ethics Grant No. 2020/EC/49.

## Conflicts of Interest

The authors declare no conflicts of interest.

## Supporting information

Supporting Information

## Data Availability

Data are available on request from the authors. The data that support the findings of this study are available from the corresponding author upon reasonable request.
