# Supplementary material for: The Political, Economic and Socio‐Cultural Discourse Surrounding the Backyard Chicken‐Rearing Farming Systems in the Western and North‐Western Provinces of Sri Lanka
Source: Vet Med Sci. 2025 Apr 15;11(3):e70174. doi: 10.1002/vms3.70174 (PMC11998949; doi:10.1002/vms3.70174)
Supplement: Supplementary file 1 — Supporting Information [file VMS3-11-e70174-s001.docx]

**Interview** **Questions**

1. **Registration**

- Have you registered as a backyard poultry farmer at the regional veterinary office?
- If yes, what benefits do you get as a registered poultry farmer?

1. **Vaccination and medication**

- What kind of medication do you give your chickens?
- Are your chickens properly vaccinated?
- How expensive are the medicines given to chickens?

1. **Economic assistance from the government / relevant authorities**

- How much initial capital did you approximately invest when starting the business?
- What sort of financial assistance do you receive from the government / relevant authorities?
- Do you have any other income-generating sources apart from the poultry business?
- Is this a profitable business? Do you earn an income from this which is substantial?

1. **Awareness**

- Explain whether you are aware of the best practices related to poultry farming.
- Have you ever attended any seminars, workshops or training programmes conducted to make the backyard poultry farmers aware of the best practices related to poultry farming?

1. **Challenges**

- What challenges/obstacles do you face as a backyard poultry farmer? (Economically, politically, socially, culturally)

1. **Diseases**

- As a farmer are you aware of the common diseases that can affect your flock?
- How do you understand that your birds are sick? Can you explain?
- So if so, as in they are sick, what will you do?
- Are you satisfied with the services provided by the veterinary officers?
- What are the most common disease conditions among your chickens?

1. **Bio-security threats**

- Are you aware of the notion of biosecurity? What does it entail or have?
- What kind of bio-security threats do you face when running a backyard poultry farm?
- Can you state some specific instances where you faced bio-security threats?
- What are the protective measures that you can implement to overcome those bio-security threats?

1. **Female empowerment**

- Discuss the amount of work contributed/shared/rendered by women in backyard farms (how the daily household work is balanced with farm work)
- Has your family applied for a business loan specifically issued for women? Did you receive assistance from the veterinary authorities to obtain the loan or any other women’s societies attached to financial institutions?
- How do women from the farming families perceive backyard poultry and their contribution? (if given) (whether they perceive this as a challenge/satisfaction or burden)
- Were the women from farming families able to save an amount from the farm income for their personal use? If yes/no how and or why?
- Are the retained earnings being used as a back-to-back investment for the farm? (Whether the income is reinvested solely for the farm or whether the profit facilitates monthly expenses of the household?)

1. **Religious and Socio-Cultural Practices Associated with Chickens**

- What are the cultural and religious practices associated with chickens in your area? Do you provide chickens from your farm for those practices?

1. **Other**

- What role does religion play in your life? Do you think religion / your beliefs play a significant role in decision-making pertaining to your profession?
- Public understanding and attitudes about backyard poultry farming?
- What are the positive/negative and or neutral attitudes of the other villagers towards farming and how do the farming families perceive it?
